# Modelling Neonatal Care Pathways for Babies Born Preterm: An Application of Multistate Modelling

**DOI:** 10.1371/journal.pone.0165202

**Published:** 2016-10-20

**Authors:** Sarah E. Seaton, Lisa Barker, Elizabeth S. Draper, Keith R. Abrams, Neena Modi, Bradley N. Manktelow, UK Neonatal Collaborative

**Affiliations:** 1 Department of Health Sciences, University of Leicester, Leicester, United Kingdom; 2 Leicester Neonatal Unit, University Hospitals of Leicester NHS Trust, Leicester, United Kingdom; 3 Neonatal Data Analysis Unit, Section of Neonatal Medicine, Department of Medicine, Imperial College, London, United Kingdom; Centre Hospitalier Universitaire Vaudois, FRANCE

## Abstract

Modelling length of stay in neonatal care is vital to inform service planning and the counselling of parents. Preterm babies, at the highest risk of mortality, can have long stays in neonatal care and require high resource use. Previous work has incorporated babies that die into length of stay estimates, but this still overlooks the levels of care required during their stay. This work incorporates all babies, and the levels of care they require, into length of stay estimates. Data were obtained from the National Neonatal Research Database for singleton babies born at 24–31 weeks gestational age discharged from a neonatal unit in England from 2011 to 2014. A Cox multistate model, adjusted for gestational age, was used to consider a baby’s two competing outcomes: death or discharge from neonatal care, whilst also considering the different levels of care required: intensive care; high dependency care and special care. The probabilities of receiving each of the levels of care, or having died or been discharged from neonatal care are presented graphically overall and adjusted for gestational age. Stacked predicted probabilities produced for each week of gestational age provide a useful tool for clinicians when counselling parents about length of stay and for commissioners when considering allocation of resources. Multistate modelling provides a useful method for describing the entire neonatal care pathway, where rates of in-unit mortality can be high. For a healthcare service focussed on costs, it is important to consider all babies that contribute towards workload, and the levels of care they require.

## Introduction

In the UK, 1 in 8 babies require specialist neonatal care after their birth[1] and the needs of these babies can vary dramatically, both in clinical approach and the length of time they require in hospital. For the most preterm babies who survive, this care can last several months or longer. Understanding the time babies spend in neonatal care is vital to aid the counselling of parents, plan care provision and ensure the appropriate funding of services. However, there are two potential problems which mean that such information has not been readily available.

First, there has been little research in modelling length of stay in neonatal care, and the research that does exist has understandably focussed on time to discharge for survivors,[2–4] or has considered all babies together irrespective of their outcome.[5] Excluding babies who die during their time in neonatal care overlooks this important group, who contribute to the workload of the health service during the time they are alive and should therefore be included in estimates of length of stay. In particular, babies born preterm have a high rate of in-hospital mortality, particularly for those born at <28 weeks gestation[6] and any analysis which is only based on survivors does not fully describe neonatal care provision or requirements. However, recent work has illustrated a statistical approach, ‘competing risks’, which can be used to appropriately include all babies when estimating the length of stay in neonatal care.[7]

Second, broad estimates of overall length of stay are of limited use as they provide no information about the type of care a baby requires during their stay in neonatal care. Within the UK, neonatal care is defined into three main levels, using criteria developed by the British Association of Perinatal Medicine (BAPM) in 2011.[8] The levels defined by BAPM are: intensive care (e.g. ventilation); high dependency care (e.g. drug infusion) or special care (e.g. phototherapy). This classification of care provision is similar to that seen in other countries.[9–11] A baby born very preterm is likely to need care provided at different levels throughout their time in hospital, and will move between these levels of care during their in-patient stay (we refer to this as the neonatal care pathway). Whilst other levels of neonatal care do exist, most notably transitional care,[12] these are not offered consistently.

Use of standard statistical approaches, which measure time until an event occurs, to measure length of stay, are unable to capture the complexity of the nature of neonatal care, whilst also modelling babies irrespective of their outcome. Recent advances such as competing risks have been used to model the length of stay for babies, and including those who die before discharge.[7] However, it is of interest to model the neonatal care pathway that a baby follows from birth, considering the different levels of care they require, before being discharged or dying in neonatal care, which previous work has not considered. Here we propose the use of a statistical method known as multistate modelling which allows the complete care pathway to be investigated for all babies: i.e. both those who survive to discharge and those who do not.

## Methods

Data were extracted from the National Neonatal Research Database (NNRD) which holds data related to the daily neonatal care, demographics and outcomes of all babies admitted to neonatal units throughout England, Wales and Scotland. The NNRD is maintained by the Neonatal Data Analysis Unit (NDAU) and created from electronic health records. Only data from England was used in the analysis of this paper because full data collection was not available for Wales and Scotland for the years analysed.

Permission was granted to use anonymised data from the NNRD for research purposes (ethics reference: 14/NW/0349, North West—Lancaster ethics committee) and agreement was obtained from all neonatal units in England (n = 162 units) that existed in 2014 to use their data for this project.

All singleton babies born at 24–31 weeks gestational age that were discharged from a neonatal unit from 2011 to 2014 were included in this analysis. Babies born prior to 24 weeks gestational age were excluded as their care relates heavily to local policies.[13] Babies were not excluded for missing or ambiguous data on any other variables. Babies who were discharged home before 34 weeks corrected age were excluded by all analyses, as this is the point at which babies begin to learn to suck and feed and a discharge home before this point is clinically implausible. Babies with a total length of stay greater than six months were also excluded as although some babies do stay in hospital this long, it is a rare occurrence and the numbers are small producing unreliable estimates. Babies were also excluded if they were discharged from neonatal care having only received intensive care or were discharged having never received special care (i.e. after receiving high dependency and potentially intensive care) as these care pathways are clinically unusual and there was insufficient statistical power to produce reliable estimates.

If a baby was not recorded as having died on a neonatal unit, they were considered to have survived to discharge. However, as the NNRD only holds data related to neonatal care, these babies might have died, or spent substantially periods of time, in other specialist services, such as paediatric wards or cardiac surgical centres. Therefore, two final outcomes were considered: death and discharge alive from the neonatal unit.

As transitional care is not offered consistently, this group has been amalgamated with special care in all analyses (the most similar level of care)[12]–to otherwise exclude this level of care would underestimate the total length of stay of many babies. In this analysis, all care is assumed to have occurred in a hierarchical manner, that is, all intensive care comes before high dependency care, and all high dependency care comes before special care. Certain transitions are clinically unlikely, although not impossible, for example, being discharged from neonatal care having only received intensive care and these are not considered in the analyses presented as the numbers experiencing them are very small and reliable estimates cannot be obtained.

### Statistical analysis

**Fig 1:**
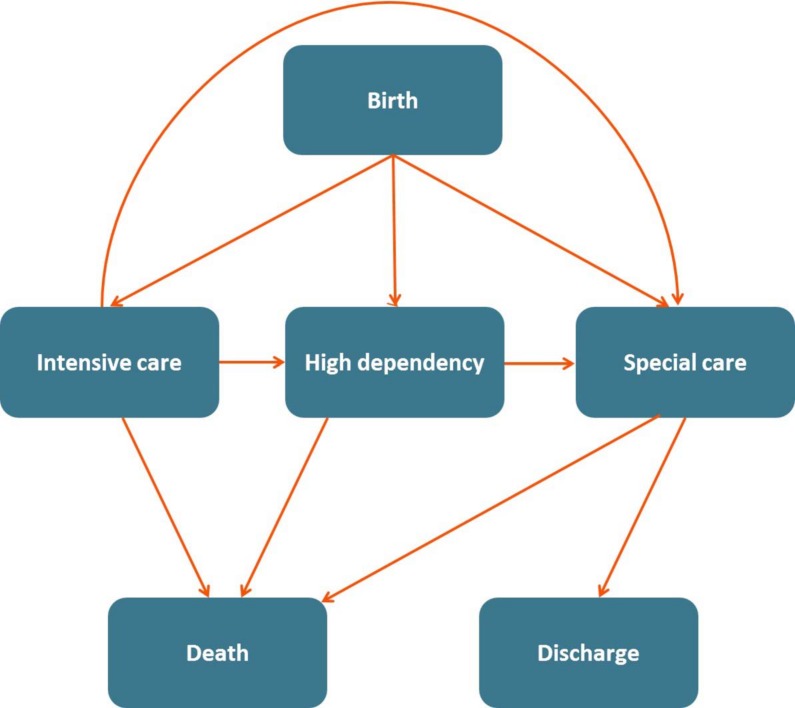
The multistate model used here to describe the neonatal care pathway. All babies begin in the birth state before following transitions (arrows) throughout the model until reaching death or discharge.

A multistate model[14] was used to describe the time period from an initial event (in this case birth) until a final endpoint (i.e. death or discharge from neonatal care) whilst also considering intermediate changes in the level of care received. The multistate model used is described in Fig 1. Each of the boxes (known as states) describes a level of care in the neonatal care pathway that a baby can potentially experience. States are ‘absorbing’ if upon entry they are impossible to exit again (i.e. death and discharge from neonatal care). Alternatively, they are known as ‘intermediate’ states if it is possible to exit them and move to a different state (i.e. the different levels of neonatal care).

The arrows denoted in Fig 1 represent ‘transitions’ a baby can make through the neonatal care pathway. Here, a baby is born and, if considered viable, admitted to the neonatal unit where they are assumed to immediately receive the highest level of care they will require during their neonatal in-patient stay, i.e. intensive care, high dependency care or special care. This is considered to be day 1 of life, so all admitted babies spend at least one day receiving neonatal care.

A Cox proportional hazards model was stratified on transition (depicted by the arrows in Fig 1) and used to estimate the transition specific hazard rates[14,15] and was used to calculate the probabilities of receiving each of the levels of care, or of having died or been discharged by any given time. These probabilities represent the observed data, and are presented as percentages in a graphical form overall without adjustment for any covariates.

The probabilities were then estimated by adjusting for week of gestational age at birth, with 27 weeks gestational age as the baseline group. The care pathway was adjusted by gestational age at birth, as this is known to be an important predictor of length of stay and mortality.[16] Every transition through the model was adjusted for gestational age as clinically it is thought to impact throughout the care pathway. The estimated probabilities of receiving each level of care, or of having died or been discharged, are presented graphically, and tabulated estimates are provided, for each week of gestational age over time since birth. Additionally, it is possible to calculate the expected length of stay[17] at each level of care for each week of gestational age by integrating the area under the probability curve, and these are provided.

The assumption of proportional hazards was tested using the Therneau-Grambsch test with p<0.001 indicating potential issues and when this occurred the Schoenfeld residuals were plotted against time to visually examine any trends.[18]

All analyses were undertaken in R 3.0.2 using the mstate command.[15,19]

## Results

There were 21,631 singleton babies born at 24–31 weeks gestational age, admitted to neonatal in-patient care in England from 2011 to 2014. The total length of stay was calculated for all babies; except those discharged home before 34 weeks corrected age (n = 205, 0.9%) or who stayed in hospital longer than six months (n = 199, 0.9%). Babies were also excluded if they were discharged from neonatal care having only received intensive care (n = 57, 0.3%) or were discharged having never received special care (i.e. after receiving high dependency and potentially intensive care, n = 132, 0.6%). A total of 21,038 babies remained in the analysis.

Summary characteristics of the included babies are provided in Table 1, which demonstrates that the population of babies discharged from neonatal care was broadly consistent throughout the years of the study. Each year, approximately 300,000 days of care were given to these babies, with the majority of this being special care.

**Table 1:** Summary statistics of babies born at 24^+0^ to 31^+6^ weeks gestational age and discharged between 2011 and 2014.

	Year of discharge/death from neonatal care			
	2011	2012	2013	2014
**Total babies admitted, n**	5,368	5,343	5,228	5,099
**Gestational age, n (%)**				
24	284 (5.3)	287 (5.4)	276 (5.3)	268 (5.3)
25	327 (6.1)	336 (6.3)	316 (6.0)	325 (6.4)
26	466 (8.6)	465 (8.7)	417 (8.0)	437 (8.6)
27	537 (10.0)	579 (10.8)	480 (9.2)	468 (9.2)
28	690 (12.9)	707 (13.2)	702 (13.4)	685 (13.4)
29	758 (14.1)	791 (14.9)	807 (15.4)	748 (14.7)
30	983 (18.3)	937 (17.5)	976 (18.7)	944 (18.5)
31	1,325 (24.7)	1,241 (23.2)	1,254 (24.0)	1,224 (24.0)
**Sex of baby**				
Male	2,953 (55.0)	2951 (55.2)	2937 (56.2)	2756 (54.1)
Female	2,411 (44.9)	2389 (44.7)	2287 (43.7)	2334 (45.8)
Indeterminate	4 (0.01)	3 (0.01)	4 (0.01)	9 (0.01)
**Total days of care**	305,150	306,267	295,828	298,177
Days of intensive care	60,995	63,040	60,348	62,058
Days of HDU	77,707	83,726	81,346	86,789
Days of special	166,448	159,501	154,134	149,330
**Birthweight (g)** Mean (SD)	1227.1 (383.2)	1215.0 (374.5)	1231.9 (378.7)	1217.2 (376.4)
**Died in neonatal care, n (%)**	492 (9.2)	487 (9.1)	416 (8.0)	367 (7.2)

**Fig 2:**
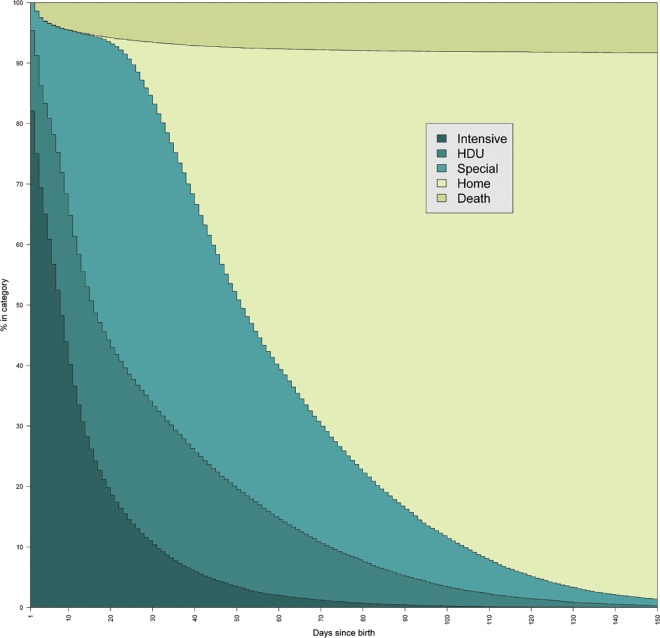
The percentage of babies receiving each level of care, or who have died or been discharged, over time.

A multistate Cox proportional hazards model, stratified for transition (the steps that can be taken through the care pathway), was fitted as depicted in Fig 1. The number of babies entering each state (the levels of care or death or discharge) is summarised in Table 2. Initially, a model was fitted with no covariates, which is presented in Fig 2. This shows the percentage of babies in each state (y-axis), at any point in time following birth (x-axis). For example, ten days after birth 40% of babies were receiving intensive care, 25% were receiving high dependency and 30% were receiving special care. At this point, no babies had been discharged from neonatal care and 5% of babies had died. These are presented as percentages as the lack of adjustment in the model means that the results are the observed data.

**Table 2:** Number of babies to enter and visit each state within the multistate model.

	To	Intensive care	High dependency	Special care	Died	Discharged
From						
Born		17,269	2,796	973	0	0
Intensive care			15,129	824	1316	0
High dependency care				17,665	260	0
Special care					186	19,276

**Fig 3:**
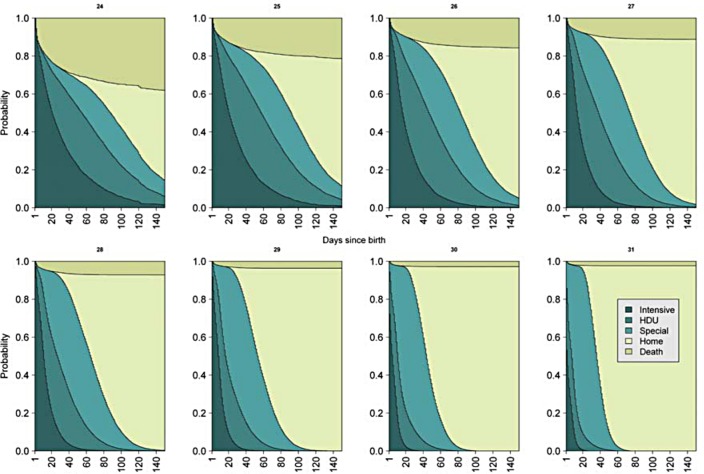
The probability of babies, adjusted for gestational age, receiving each level of care, or who have died or been discharged, over time.

Stacked plots were produced adjusted for each week of gestational age in order to show the probability of receiving any level of care, or of having died or being discharged over time (Fig 3). As week of gestation at birth increased the probability of mortality decreased and the need for higher levels of care also decreased. As expected, there was an inverse relationship between time of discharge home and gestational age. For example, for babies born at 24 weeks, at seven days of life the probability of receiving intensive care was 0.77; high dependency was 0.07; special care was 0.01 and the probability of having died was 0.15. Conversely, for babies born at 31 weeks, at seven days of life the probability of receiving intensive care was much lower at 0.20; high dependency was higher at 0.29; special care was 0.49. The probability of death for this group was much lower at seven days: 0.02. Results related to the babies with the least data, i.e. those born at 24 and 25 weeks gestational age should be interpreted cautiously.

From Fig 3, the probabilities of receiving each level of care, or of having died or being discharged can be estimated. Tables 3 and 4 provide the probabilities at selected time points for babies born at 24 weeks and 31 weeks.

**Table 3:** Probabilities of 24 week babies receiving each level of care or who have died or been discharged estimated from the multistate model with an adjustment for gestational age.

Day following birth	Intensive care	High dependency	Special care	Discharge	Death
1	1.0	0.0	0.0	0.0	0.0
2	0.932	0.012	0.0	0.0	0.055
3	0.879	0.023	0.001	0.0	0.096
4	0.845	0.032	0.002	0.0	0.120
5	0.821	0.043	0.003	0.0	0.133
6	0.797	0.054	0.004	0.0	0.145
7	0.775	0.066	0.005	0.0	0.154
10	0.706	0.105	0.013	0.0	0.176
14	0.616	0.157	0.027	0.0	0.200
30	0.380	0.276	0.077	0.002	0.265
50	0.218	0.310	0.146	0.023	0.303
100	0.057	0.162	0.198	0.232	0.349
150	0.015	0.044	0.082	0.477	0.380

**Table 4:** Probabilities of 31 week babies in each level of care or who have died or been discharged estimated from the multistate model with a categorical term for gestational age. Note that everyone has at least one day of care.

Day following birth	Intensive care	High dependency	Special care	Discharge	Death
1	0.533	0.324	0.144	0.0	0.0
2	0.449	0.330	0.216	0.0	0.006
3	0.383	0.300	0.306	0.001	0.011
4	0.334	0.296	0.356	0.001	0.014
5	0.288	0.298	0.398	0.002	0.015
6	0.244	0.296	0.443	0.002	0.016
7	0.200	0.290	0.493	0.002	0.017
10	0.089	0.236	0.654	0.003	0.019
14	0.027	0.151	0.797	0.005	0.021
30	0.0	0.038	0.671	0.269	0.023
50	0.0	0.005	0.094	0.878	0.024
100	0.0	0.0	0.0	0.977	0.024
150	0.0	0.0	0.0	0.977	0.024

The expected time spent in each state can be calculated for all babies, irrespective of their outcome, born at each week of gestational age and these are provided in Table 5.

**Table 5:** Expected time spent receiving each level of care, and total length of stay, by gestational age. Results are rounded up to the nearest day.

Gestational age	Intensive care (days)	High dependency (days)	Special care (days)	Total (days)
24	33	29	22	84
25	30	33	27	90
26	23	29	30	82
27	18	25	31	74
28	14	19	33	66
29	10	13	33	56
30	6	9	31	46
31	4	6	28	38

In addition to transition probabilities, it is also possible to estimate hazard ratios for each week of gestation as gestational age was modelled categorically (Table 6). Each hazard ratio can be interpreted as the hazard of experiencing that transition through the neonatal care pathway, for a given week of gestational age at birth, compared to the babies who make that transition who were born at 27 weeks. For example, the probability of death at any time point, i.e. the hazard, after receiving only intensive care is three times higher in the 24 weeks group compared to the 27 weeks (Hazard Ratio: 3.03, 95% CI: 2.51 to 3.65, p<0.001). Conversely, the hazard ratio of experiencing the same transition in the 31 weeks group compared to 27 weeks was 0.64 (95% CI: 0.49 to 0.84, p = 0.001), suggested a reduced hazard of death after receiving only intensive care in the 31 week babies compared to the 27 week babies.

**Table 6:** Hazard ratios of each transition by gestational age. The hazard ratio compares back to the baseline of 27 weeks gestational age for that transition.

Gestational age Transition	Hazard ratio	95% Confidence Interval	p-value
**24 weeks**			
IC -> HD	0.38	0.35 to 0.41	<0.01
IC -> SC	0.65	0.26 to 1.62	0.36
IC -> Death	3.03	2.51 to 3.65	<0.01
HD -> SC	0.51	0.47 to 0.56	<0.01
HD -> Death	1.85	1.21 to 2.82	0.01
SC -> Home	0.58	0.53 to 0.64	<0.01
SC -> Death	4.51	2.15 to 9.45	<0.01
**25 weeks**			
IC -> HD	0.52	0.48 to 0.56	<0.01
IC -> SC	0.25	0.07 to 0.88	0.03
IC -> Death	1.63	1.33 to 1.99	<0.01
HD -> SC	0.61	0.57 to 0.66	<0.01
HD -> Death	1.05	0.68 to 1.61	0.84
SC -> Home	0.69	0.64 to 0.75	<0.01
SC -> Death	3.40	1.73 to 6.71	<0.01
**26 weeks**			
IC -> HD	0.75	0.70 to 0.80	<0.01
IC -> SC	0.52	0.23 to 1.32	0.18
IC -> Death	1.25	1.02 to 1.53	0.03
HD -> SC	0.80	0.75 to 0.86	<0.01
HD -> Death	0.88	0.58 to 1.34	0.55
SC -> Home	0.80	0.75 to 0.86	<0.01
SC -> Death	2.96	1.68 to 5.21	<0.01
**27 weeks**			
IC -> HD	Reference	Reference	Reference
IC -> SC	Reference	Reference	Reference
IC -> Death	Reference	Reference	Reference
HD -> SC	Reference	Reference	Reference
HD -> Death	Reference	Reference	Reference
SC -> Home	Reference	Reference	Reference
SC -> Death	Reference	Reference	Reference
**28 weeks**			
IC -> HD	1.38	1.30 to 1.47	<0.01
IC -> SC	3.95	2.21 to 7.07	<0.01
IC -> Death	0.71	0.57 to 0.87	<0.01
HD -> SC	1.40	1.32 to 1.48	<0.01
HD -> Death	0.79	0.51 to 1.22	0.29
SC -> Home	1.25	1.18 to 1.33	<0.01
SC -> Death	0.59	0.31 to 1.04	0.07
**29 weeks**			
IC -> HD	2.04	1.92 to 2.17	<0.01
IC -> SC	9.93	5.71 to 17.3	<0.01
IC -> Death	0.44	0.34 to 0.57	<0.01
HD -> SC	1.97	1.85 to 2.09	<0.01
HD -> Death	0.49	0.28 to 0.86	0.01
SC -> Home	1.87	1.76 to 1.98	<0.01
SC -> Death	0.27	0.15 to 0.52	<0.01
**30 weeks**			
IC -> HD	2.86	2.69 to 3.04	<0.01
IC -> SC	26.7	15.6 to 45.9	<0.01
IC -> Death	0.55	0.42 to 0.72	<0.01
HD -> SC	2.44	2.30 to 2.60	<0.01
HD -> Death	0.26	0.12 to 0.55	<0.01
SC -> Home	3.17	2.99 to 3.36	<0.01
SC -> Death	0.16	0.08 to 0.30	<0.01
**31 weeks**			
IC -> HD	3.48	3.27 to 3.70	<0.01
IC -> SC	53.6	31.4 to 91.6	<0.01
IC -> Death	0.64	0.49 to 0.84	<0.01
HD -> SC	3.10	2.91 to 3.30	<0.01
HD -> Death	0.48	0.25 to 0.95	0.03
SC -> Home	6.26	5.90 to 6.63	<0.01
SC -> Death	0.10	0.05 to 0.20	<0.01

In this table the following acronyms are used: intensive care (IC); high dependency care (HD) and special care (SC)

Certain transitions should be interpreted with caution, as lack of data means hazard ratios are potentially poorly estimated. For example, very few babies receive intensive care and then special care, with no high dependency care, and this is seen in the hazard ratio of this transition for babies born at 31 weeks which is estimated as being 53.6 (95% CI: 31.4 to 91.6) with a wide confidence interval.

Two transitions were identified as potentially breaching the proportional hazards assumption: intensive care to high dependency and high dependency to special care. This is unsurprising as these transitions had the most data, and potentially needed to be modelled in a more complex manner, such as via using fractional polynomials[20] or time varying covariates. However, examination of the Schoenfeld residuals against a function of time indicated that the issues with these transitions were likely to be minimal, but future work will investigate this further. Further issues of proportional hazards may also exist in other transitions, most notably the transition into discharge.

## Discussion

In this paper an alternative statistical approach has been proposed and illustrated in order to investigate length of neonatal in-patient stay, which allows the incorporation of babies that died in neonatal care, whilst also considering the levels of care that all babies require. Previous work investigating length of stay has either removed babies that die[2–4] or included them inappropriately, potentially biasing the results.[5] The modelling of survival and length of stay is becoming increasingly important in neonatal care, particularly as survival at early gestational ages improves and the care requirements of these babies increases.[21] It might seem appropriate to focus attention on survivors, as the resource use and workload they require is great. However, this does not portray an accurate picture of the total workload for neonatal care as for the most preterm babies mortality rates are high.[4,21]

Although previous work[7] has considered babies that die during their time in neonatal care, babies born very preterm can spend a long time in hospital and receive complex types of care.[4,7] Estimating total length of stay alone does not provide a clear picture of the experiences they have during their neonatal stay. Detailed information about neonatal care is important for counselling families and for commissioning services.

The probabilities of receiving each level of care, or of having died or being discharged presented here can be used in the counselling of parents and informing the conversations had with clinicians. For example, nearly all 31 week babies were discharged or had died by 50 days after birth. Conversely, for babies born at 24 weeks, there was a small probability of remaining in hospital even 150 days after birth. Future work can provide detailed estimates for clinicians to use in the counselling of parents.

For a healthcare service increasingly focussed on costs, estimating detailed information about length of stay aids the informing of neonatal service provision, commissioning and funding. Here we estimated the expected time receiving each level of care for all babies, irrespective of outcome. These estimates could be used to aid the commissioning of resources by calculating the expected number of babies at each gestation and multiplying by the expected days of care to receive an estimate of the resource need.

The results presented here arise from use of multistate modelling[14,22] which is a relatively novel approach that has been used in other medical areas (e.g. cancer[23]). User-friendly software has now made implementation of this method more straightforward.[15,19] Multistate modelling allows consideration of multiple ‘competing’ outcomes (where the occurrence of one outcome prevents the occurrence of others), whilst also modelling intermediate steps (here levels of care). This allows for modelling of different levels of care, as well as death and discharge over time. Here the results are presented graphically, for ease of interpretation and in the future these plots or similar will provide a useful aide for clinicians giving information about the length of time spent at each level of care, whilst also considering the probability of surviving to discharge.

The data used here cover all admissions and discharges from English neonatal units, avoiding the bias that would be introduced by examining an individual hospital, and thus be subject to local policy and practice.[13] Whilst population differences will exist between individual units, our aim was to provide a population-based estimate, and future work could examine individual hospitals or Neonatal Networks to investigate variations within England.

### Strengths and limitations

A strength of this work is that this is the first time that the entire neonatal care pathway has been described, using national data, whilst simultaneously considering the different levels of care and competing outcomes of death or discharge from neonatal care. However, many people, criticise the Cox model, which was used here, as having been overused when other methods would be more appropriate.[24] However, we did not want to impose distributional assumptions on the shape of the hazard function by using a parametric approach as examination of the observed hazards indicated this might be inappropriate.

There are issues of proportional hazards in at least two of the transitions but it is well known that the introduction of time dependent effects in Cox modelling is difficult and computationally intensive particularly with large datasets such as this one.[25] Future work will need to investigate alternative methods, including a flexible parametric approach[26] to allow further flexibility in all transitions. Additionally, the lack of data in the group of babies born at 24 and 25 weeks gestational age means the model here does not fit as well to these groups. This is particularly apparent when considering the time at which the probability of discharge occurs in the extremely preterm babies, and these results should be interpreted cautiously and investigated further.

Gestational age was modelled categorically and some would argue this may lead to a loss of statistical power.[27] However, there is an underlying clinical meaning to completed weeks of gestation which is often used in practice and allows for simple interpretation of the hazard ratios.[28] Other covariates including birthweight are likely to be important adjustments to make in this work[29] and future research will consider their inclusion, as here the aim was to provide an introduction to this type of analysis. Similarly, this work has considered singletons, and multiples are known to comprise a large proportion of the preterm population. Future work will include multiples and consider appropriate stratification or adjustment of the methods presented here. Adjustments for covariates in a multistate analysis should be made with care to avoid overfitting, particularly where the number of events for a particular transition is small. Gestational age was adjusted for here on every transition, but adjustments can be made which are assumed to be the same for all transitions.

Only deaths which occurred in neonatal care are considered here, and deaths may have occurred elsewhere. However, it was not possible to retrieve this information. Future work will link the neonatal care with care received elsewhere. However, we believe the number of deaths in other locations is likely to be a small number.

Finally, care here was assumed to have occurred in a hierarchical manner, with all intensive care preceding high dependency, and all high dependency care preceding special care. Whilst this is unlikely to be the reality for some babies, particularly those that become more unwell during their time in hospital (e.g. contracting sepsis), this work still provides a useful resource for clinicians and the commissioning of services. Future work will investigate the order in which babies receive their differing levels of care.

## Conclusion

Modelling of survival and length of stay has become increasingly important in neonatal care to provide parents with accurate and realistic information about their baby’s care. It is also important for the commissioning of services and resources. Multistate modelling is a statistical approach which begins to describe the neonatal care pathway in a more complete way than has been previously undertaken. These methods allow for modelling of mortality and survival, whilst also considering all the potential levels of care which can occur along the care pathway. Estimates of the probability of being in hospital, receiving a given level of care, or of having died or been discharged can be produced and used to inform conversations between clinicians and parents. Future research should refine and improve these estimates particularly for the extremely preterm babies.
